# Perioperative parameters-based prediction model for acute kidney injury in Chinese population following valvular surgery

**DOI:** 10.3389/fcvm.2023.1094997

**Published:** 2023-03-07

**Authors:** Yun Yan, Hairong Gong, Jie Hu, Di Wu, Ziyu Zheng, Lini Wang, Chong Lei

**Affiliations:** ^1^ Department of Anesthesiology and Perioperative Medicine, Xijing Hospital, The Fourth Military Medical University, Xi’an, China; ^2^ Department of Critical Care Medicine, the First Medical Centre, Chinese PLA General Hospital, Beijing, China; ^3^ Department of School of Biological Science and Medical Engineering, Beihang University, Beijing, China

**Keywords:** lasso logistics regression, machine learning, cardiac surgery, acute kidney injury, prediction

## Abstract

**Background:**

Acute kidney injury (AKI) is a relevant complication after cardiac surgery and is associated with significant morbidity and mortality. Existing risk prediction tools have certain limitations and perform poorly in the Chinese population. We aimed to develop prediction models for AKI after valvular cardiac surgery in the Chinese population.

**Methods:**

Models were developed from a retrospective cohort of patients undergoing valve surgery from December 2013 to November 2018. Three models were developed to predict all-stage, or moderate to severe AKI, as diagnosed according to Kidney Disease: Improving Global Outcomes (KDIGO) based on patient characteristics and perioperative variables. Models were developed based on lasso logistics regression (LLR), random forest (RF), and extreme gradient boosting (XGboost). The accuracy was compared among three models and against the previously published reference AKICS score.

**Results:**

A total of 3,392 patients (mean [SD] age, 50.1 [11.3] years; 1787 [52.7%] male) were identified during the study period. The development of AKI was recorded in 50.5% of patients undergoing valve surgery. In the internal validation testing set, the LLR model marginally improved discrimination (C statistic, 0.7; 95% CI, 0.66–0.73) compared with two machine learning models, RF (C statistic, 0.69; 95% CI, 0.65–0.72) and XGBoost (C statistic, 0.66; 95% CI, 0.63–0.70). A better calibration was also found in the LLR, with a greater net benefit, especially for the higher probabilities as indicated in the decision curve analysis. All three newly developed models outperformed the reference AKICS score.

**Conclusion:**

Among the Chinese population undergoing CPB-assisted valvular cardiac surgery, prediction models based on perioperative variables were developed. The LLR model demonstrated the best predictive performance was selected for predicting all-stage AKI after surgery.

**Clinical trial registration:**

Trial registration: Clinicaltrials.gov, NCT04237636.

## Introduction

Acute kidney injury (AKI) is one of the most common complications following cardiac surgery, with documented incidence ranging from 5% to 54% (1, 2). The occurrence of cardiac surgery-associated AKI (CSA-AKI) is associated with an increase in both short- and long-term adverse outcomes, as well as healthcare expenses (3, 4). Approximately 25% of patients with AKI will advance to chronic kidney disease (CKD) after three years (5). And the 5-year and 7-year cohort survival rates of patients with severe AKI was 54% and 38%, respectively (6). The corresponding annual cost for CSA-AKI was almost $1 billion in the United States (7). Currently, no treatment for CSA-AKI has been proven to be effective in large-scale clinical trials (1). Therefore, the management of postoperative CSA-AKI is now primarily focused on prevention, making screening and identification of high-risk patients of paramount importance.

The effectiveness of screening and identifying high-risk patients is contingent upon the development of easy-use, accurate, and high-performance prediction models. Unfortunately, currently available risk prediction models are unsuitable for widespread clinical use due to intrinsic defects, and there has been no significant improvement in early diagnosis (8). First, the majority of established prediction models were developed to classify patients with a high risk of acute renal failure (ARF) and/or a requirement for renal replacement therapy (RRT), but with varying definitions of ARF or RRT (9–13). ARF or RRT are clinically consistent with advanced or end-stage kidney injury, and such severe kidney damage was typically irreversible. Thus, identifying patients with a high risk of early-stage CSA-AKI and initiating treatment was warranted. Second, in the majority of previous models, only preoperative risk factors were included, whereas intra- and postoperative risk predictors were neglected (14), resulting in unsatisfactory prediction performance. Third, the consensus KDIGO (Kidney Disease: Improving Global Outcomes) definition of AKI, which allows for the classification of AKI by the severity of kidney injury from mild to severe (9), with higher sensitivity to predict AKI-related morbidity and mortality (15), was not implemented in the majority of prediction models or screen scores, such as the widely used Cleveland Clinical score (11). Finally, using conventional logistic regression to construct prediction models of CSA-AKI necessitates meeting specific assumptions, whereas machine learning techniques can model nonlinear relationships and interactions, as well as handle large numbers of input features (16). Both classical regression and machine learning can be used to construct prediction models, with advantages and disadvantages depending on the clinical scenario and data attributes.

It has been demonstrated that the risk of postoperative AKI in patients undergoing valve procedures is higher than in those undergoing coronary artery bypass grafting (CABG) surgery (17). Undergoing valve surgery is an independent risk factor for postoperative ARF (18). Most of the above-mentioned models have concentrated on all cardiac surgeries or those specific to CABG surgery, resulting in poor accuracy in predicting the risk of postoperative CSA-AKI for cardiopulmonary bypass (CPB) assisted open-heart valvular procedures.

The current study aimed to investigate the utilization of pre-, intra-, and early postoperative clinical features to predict mild or moderate to severe CSA-AKI in patients undergoing CPB-assisted valvular surgery. This study compared the prediction accuracy of models developed by traditional logistic regression with machine learning techniques, including random forest (RF) and eXtreme Gradient Boosting (XGboost). The models with the best performance were then chosen for future clinical use.

## Methods

### Study design, setting and participants

The study followed the TRIPOD (Transparent Reporting of a multivariable prediction model for Individual Prognosis Or Diagnosis) statement (19) for prediction model development and validation. The institutional review board of Xijing Hospital reviewed and approved (KY20192157-C-1) the protocol of this study and exempted the requirement for obtaining informed consent due to the retrospective, minimal-risk nature of the study. This project was registered on clinicaltrials.gov: NCT04237636.

The cardiovascular surgery cohort from December 2013 to November 2018 in Xijing hospital was used for prediction model derivation and validation. Surgical and perioperative management data were obtained from the Anesthesiology Registry system and electronic health records system for all patients who underwent CPB-assisted valve surgery. Of note, only the first procedure record was included in the analyses for patients with multiple eligible procedures during the study period.

The study included all adult patients (18 years and older) who underwent elective valvular surgery. Patients who underwent off-pump coronary surgery; or had a history of valvular surgery; or with pre-existing renal failure (defined as preoperative serum creatinine greater than 4.0 mg/dl or higher, or receiving renal replacement therapy within 48 h of serum creatinine measurement; with an estimated glomerular filtration rate (eGFR) less than <30 ml/min/1.73 m^2^; or required preoperative dialysis) were excluded. Patients without baseline or postoperative serum creatinine measurements were excluded from the analysis.

### Data collection and predictors

Perioperative patients and procedure-related variables were chosen for modeling. On the basis of available literature and clinical expertise, a set of predictors was assessed. These include patient demographics, comorbidities, medical history, preoperative medications and the use of an intra-aortic balloon pump (IABP), laboratory tests, procedure-related variables, and early postoperative variables (detailed candidate variables were listed in Additional File S1). A total of 48 variables were obtained and the final prediction models were developed based on predictors derived from the above-mentioned candidate variables.

### Outcome measures

The study used modified KDIGO criteria to diagnose CSA-AKI by using the most recent preoperative serum creatinine measurement as the baseline. In brief, stage 1 AKI was defined as an increase of serum creatinine ≥26.5 mmol L^−1^ within 48 h or a 1.5–1.99 fold elevation within seven days after surgery, while stage 2 AKI was defined as a 2.0–2.99 fold increase, and stage 3 AKI defined as a ≥ 3.0 times increase or creatinine level >354 mmol L^−1^ or the initiation of dialysis (20). The primary outcome was the development of any-stage postoperative AKI. A set of models to predict stage 2 or stage 3 AKI (moderate to severe AKI) were also developed as a secondary analysis.

### Modeling strategies

Two sets of models were built. One for predicting all-stage AKI, and the other for moderate to severe AKI (stages 2 and 3 AKI).

To develop models for predicting all-stage AKI, the data were randomly split into 2 subsets: 70% for model development and 30% for model testing. Since the incidence of stages 2 and 3 AKI (moderate to severe injury) was relatively low with an incidence of 7.4% in the full cohort, a complete dataset was used to develop models for predicting moderate to severe AKI.

Models were developed with traditional logistic regression and machine learning techniques: (1) logistic regression was combined with the least absolute shrinkage and selection operator (LASSO) regularization. LASSO is effective in variable selection and parameter elimination by using shrinkage. LASSO added the L1 norm of the feature coefficients as a penalty term to the loss function, and thus forced the coefficients of those weak features to become zero. After abandoning those redundant features, 14 selected features were left for model construction (Appendix Figure S1). (2) eXtreme Gradient Boosting (XGboost) (21), a kind of gradient descent boosting. It develops an interpretable model using a sequence of decision trees to make predictions. The advantages of XGboost lie in its ability to account for higher-order interactions and complex nonlinear relationships between variables and outcomes; (3) random forests (RF), a learning method for classification. They generate reasonable predictions across a wide range of data with minimal configuration. The two machine learning models, XGboost, and RF were fitted with ten-fold cross-validation to fine-tune the model parameters.

### Statistical analysis

The estimation of overall outcome prevalence in the target population, the number of candidate predictors, and the predicted model performance regarding the overall model fit (R^2^) (22) were used to determine the sample size for binary outcome prediction model development. We did not calculate the sample size in advance because we utilized all accessible data throughout the study period. However, we did a *post hoc* sample size calculation to verify whether the developed models ensure accurate prediction. Selecting an estimated C statistic (AUC) of 0.75, a prevalence of all-stage AKI of 50% for the patient undergoing valvular surgery, and a candidate predictor of 40, model development required at least 1,739 cases. In the training subset of this study, a sample size of 2,375 (70% of the whole cohort) was sufficient to ensure precise predictions and minimize over-fitting. Using an incidence of moderate to severe AKI of 8%, as indicated by the available data in the current study, at least 5,586 cases were required to develop a model for predicting stages 2 or 3 AKI (calculated by R package pmsampsize). Therefore, this subset of models was under-powered. However, the ten events per predictor degree of freedom rule were followed to reduce over-fitting.

Descriptive statistics were reported as median (IQR) or mean (SD) for continuous variables as appropriate, and as frequency or percentage for categorical variables. Mann–Whitney *U* test or chi^2^ test was used for the comparison of continuous or categorical variables, respectively. Missing data were either omitted (percentage of missing data less than 5%) or imputed (proportion of missing greater than or equal to 5%) according to the percentage of missing. Using the random forest (RF) approach, the missing entries were inputted. RF was able to deal with a mixture of continuous and categorical variables when imputing missing values. During the imputation, the original dataset was divided into two parts according to whether the variable is observed or missing in the original dataset. The observed data were used as the training set, and the missing observations were used as the prediction set. The missing part of the variable under imputation is replaced by predictions from RF models.

Model discrimination was assessed with concordance statistic (C-statistic), also known as areas under the (AUC) receiver operating characteristic curve (ROC). Additionally, the predictive performance of the models was evaluated based on their sensitivity, specificity, positive predictive value (PPV), and negative predictive value (NPV), as well as the F1 score. Calibration was graphically assessed by plotting the risk of AKI development against observed risk, with a perfectly calibrated prediction lying on the 45-degree line. The calibration plots represent the model confidence. The models of all-stage AKI were internally validated and calibrated using bootstrapping of the training set for 1,000 resampling iterations and bootstrapping of the testing set for 100 resampling iterations, respectively. The constructed models were compared with the previously published reference models. For the model to predict any stage AKI, the reference model was the AKICS score (23); while for the model to predict moderate to severe AKI (stage 2 or 3 AKI), the Cleveland Clinical score was used as the reference model (11). Integrated discrimination improvement (IDI) and net reclassification improvement (NRI) were used to calculate the performance improvement of the constructed models over the reference models. Decision curve analysis was used to compute the net benefit of decisions, so as to evaluate the clinical applicability of the different constructed models. In addition, to improve the interpretability of the constructed models, especially for the machine learning algorithm, we adopted the Shapley Additive exPlanation (SHAP) method to demonstrate the importance of each variable. SHAP values were calculated to provide accurate attribution values for each feature within each prediction model (14). We also calculated the variance inflation factor of the variables from the model with the best performance.

A nomogram was finally constructed based on the variables from the model with the best performance, aka the LASSO logistic regression model in the current study. An online calculator representing the regression equation of the final model was provided at https://anun.shinyapps.io/AKI-prediction/.

Reported statistical significance levels were all 2-sided, *P* < .05. All data analyses were developed in Python Software (version 3.9, https://www.python.org) with related packages (pandas, numpy, scikit-learn, matplotlib) and R software (Version 4.2.1, https://www.r-project.org) with related relevant packages (tableone, rms, ROCR, glmnet, pROC, Hmisc, DynNom, shiny, pmsampsize, regplot, car).

## Results

### Characteristics of study population

The medical records of 3,494 patients from December 2013 to November 2018 at Xijing hospital were reviewed and identified. After excluding 102 patients, 3,392 patients were included in the final analysis (Appendix Figure S2). The training set consisted of 2,374 patients while the testing set consisted of 1,018 patients. The mean (SD) age in the whole study population was 50.1 (11.3) years, 1,787 participants (52.7%) were men. The mean (SD) age in the training set was 50.1 (11.2) years; 1,240 participants (52.2%) were men. In the testing set, the mean (SD) age was 49.9 (11.4) years, and 547 (71%) were men. Among 3,392 patients in the primary cohort, AKI, as per KDIGO criteria, was observed in 1,713 patients (50.5%), with 1,175 (49.5%) patients in the training set and 538 (52.8%) patients in the testing set developed all stage AKI. The incidences of stage 2 or 3 AKI were 166 (7%) and 87 (8.5%) in the training and testing set, respectively. The variables between the training set and testing set were well balanced as demonstrated in Table 1. A total of 786 (23.2%) patients had hypertension, 452 (13.3%) with pulmonary hypertension, and 1,158 (34.1%) experienced atrial fibrillation. In the study cohort, 3,294 (97.1%) patients underwent a valvular procedure, while the remaining 2.9% of patients underwent combined valvular and CABG procedures.

**Table 1:** Patient characteristics.

Characteristic	All	Training set	Testing set	*p* value
Patient population, *n*	3392	2374	1018	
**Demographic data**				
Age, mean (SD)	50.1 (11.3)	50.1 (11.2)	49.9 (11.4)	0.59
Male, *n* (%)	1,787 (52.7)	1,240 (52.2)	547 (53.7)	0.44
BMI, mean (SD)	22.9 (3.2)	22.9 (3.1)	23.0 (3.3)	0.62
Smoking, *n* (%)	303 (8.9)	222 (9.4)	81 (8.0)	0.21
Drinking, *n* (%)	144 (4.2)	102 (4.3)	42 (4.1)	0.89
**Surgery type**				0.27
Valve surgery only, *n* (%)	3,294 (97.1)	2,300 (96.9)	994 (97.6)	
Valve and CABG surgery, *n* (%)	98 (2.9)	74 (3.1)	24 (2.4)	
**Medical history**				
Angina, *n* (%)	55(1.6)	38(2.6)	17(1.7)	0.68
Stroke, *n* (%)	71 (2.1)	52 (2.2)	19 (1.9)	0.41
COPD, *n* (%)	51 (1.5)	38 (1.6)	13 (1.3)	0.57
Hypertension, *n* (%)	786 (23.2)	550 (23.2)	236 (23.2)	0.97
Arrythmia, *n* (%)	1,214 (35.8)	844 (35.6)	370 (36.3)	0.68
Diabetes, *n* (%)	11 (0.3)	7 (0.3)	4 (0.4)	0.96
Liver Disease, *n* (%)	16 (0.5)	13 (0.5)	3 (0.3)	0.42
MI, *n* (%)	3 (0.1)	2 (0.1)	1 (0.1)	1
Atrial fibrillation, *n* (%)	1,158 (34.1)	808 (34.0)	350 (34.4)	0.87
Pulmonary hypertension, *n* (%)	452 (13.3)	322 (13.6)	130 (12.8)	0.57
NYHA, *n* (%)				0.44
Class I	4 (0.1)	2 (0.1)	2 (0.2)	
Class II	603 (17.9)	436 (18.5)	167 (16.5)	
Class III	2,626 (78.1)	1,824 (77.6)	802 (79.4)	
Class IV	129 (3.8)	90 (3.8)	39 (3.9)	
EF (%), mean (SD)	50 (10)	50 (10)	50 (10)	0.43
**Cardiac surgery history, *n* (%)**				0.15
Open heart	86 (2.5)	53 (2.2)	33 (3.2)	
Percutaneous intervention	65 (1.9)	49 (2.1)	16 (1.6)	
**Diagnosis, *n* (%)**				0.86
Congenital valve disease	1,513 (44.6)	1,065 (44.9)	448 (44.0)	
Infectious endocarditis	199 (5.9)	137 (5.8)	62 (6.1)	
Rheumatic valve disease	1,680 (49.5)	1,172 (49.4)	508 (49.9)	
**Preoperative medications**				
Contrast, *n* (%)	481 (14.2)	351 (14.8)	130 (12.8)	0.13
Nephrotoxic antibiotics, *n* (%)	43 (1.3)	29 (1.2)	14 (1.4)	0.84
ACEI, *n* (%)	35 (1.0)	26 (1.1)	9 (0.9)	0.71
ARB, *n* (%)	38 (1.1)	26 (1.1)	12 (1.2)	0.97
Aspirin, *n* (%)	49 (1.4)	37 (1.6)	12 (1.2)	0.48
Levosimendan, *n* (%)	300 (8.8)	220 (9.3)	80 (7.9)	0.20
NSAIDs, *n* (%)	49 (1.4)	37 (1.6)	12 (1.2)	0.48
IABP, *n* (%)	3 (0.1)	3 (0.1)	0 (0)	0.55
**Baseline laboratory findings**				
eGFR, mean (SD)	71.6 (14.8)	71.4 (14.8)	72.2 (14.9)	0.15
Serum creatinine, mean (SD)	96.2 (17.4)	96.2 (16.7)	96.3 (18.6)	0.38
Uric acid, mean (SD)	294.5 (99.3)	295.6 (100.4)	292.0 (96.8)	0.33
Hemoglobin, mean (SD)	139.0 (20.0)	139.1 (20.0)	138.9 (20.0)	0.78
WBC, mean (SD)	6.4 (2.0)	6.4 (2.1)	6.4 (1.9)	0.87
Albumin, mean (SD)	42.2 (4.7)	42.3 (4.7)	42.0 (4.7)	0.05
Total bilirubin, mean (SD)	17.6 (10.2)	17.6 (10.2)	17.6 (10.1)	0.87
Prothrombin, mean (SD)	11.8 (2.6)	11.7 (2.6)	11.8 (2.6)	0.54
**Intraoperative variables**				
Duration of CPB, mean (SD)	125.6 (46.4)	125.2 (46.5)	126.6 (46.2)	0.42
Duration of cross aortic clamping, mean (SD)	69.2 (35.0)	69.0 (29.2)	69.9 (45.8)	0.56
Glucose, mean (SD)	7.6 (2.1)	7.6 (2.1)	7.5 (2.0)	0.78
Hematocrit, mean (SD)	23.9 (4.5)	23.8 (4.5)	24.1 (4.5)	0.07
Temperature, mean (SD)	30.9 (1.3)	30.9 (1.4)	30.9 (1.3)	0.53
Volume of hydroxyethyl starch, mean (SD)	18.2 (125.1)	18.8 (137.3)	16.7 (90.6)	0.58
Autologous blood transfusion, mean (SD)	604.2 (251.5)	605.5 (246.8)	601.1 (262.1)	0.64
Fluid balance during CPB, mean (SD)	−41.8 (765.1)	−28.4 (759.2)	−73.2 (778.2)	0.12
Total fluid, mean (SD)	626.4 (949.7)	643.4 (967.0)	586.7 (907.5)	0.10
VIS, mean (SD)	6.1 (3.4)	6.0 (3.3)	6.3 (3.6)	0.01
Lactate, mean (SD)	5.2 (2.5)	5.1 (2.5)	5.2 (2.5)	0.51
**Postoperative variables**				
CVP at ICU admission, mean (SD)	7.09 (2.31)	7.08 (2.28)	7.11 (2.38)	0.22

Glucose and lactate were the highest level during CPB, hematocrit and temperature were the lowest level during CPB, autologous blood transfusion was the volume during the CPB. BMI, body mass index; CABG, coronary artery bypass grafting; COPD, chronic obstructive pulmonary disease; MI, myocardial infarction; NYHA, New York heart association; ACEI, angiotensin-converting-enzyme inhibitor; ARB, angiotensin II receptor blocker; NSAIDs, non-steroidal anti-inflammatory drugs; IABP, intra-aortic balloon pump; eGFR, estimated glomerular filtration rate; WBC, white blood cell; EF, ejection fraction; CPB, cardiopulmonary bypass; VIS, vasoactive inotropic score; CVP, central venous pressure.

### Features selected by LASSO

Among 48 features extracted from EHRs, including 25 categorical features and 23 continuous features that underwent feature selection by the LASSO, 14 features were finally selected for modeling (Appendix Figure S1). The variables finally included in the lasso logistic regression model included age, gender, hemoglobin, pulmonary hypertension, arrhythmia, hypertension, duration of CPB, the volume of autologous blood transfusion, pre-operative diagnosis, the highest level of lactate during the operation, albumin, BMI, vasoactive inotropic score and ejection fraction. The postoperative variable of central venous pressure (CVP) at the time of ICU admission was investigated as a potential feature in model development. However, CVP at ICU admission was omitted from the final model due to the shrinkage of this variable's coefficient *via* lasso regression.

### Model performance

**Figure 1:**
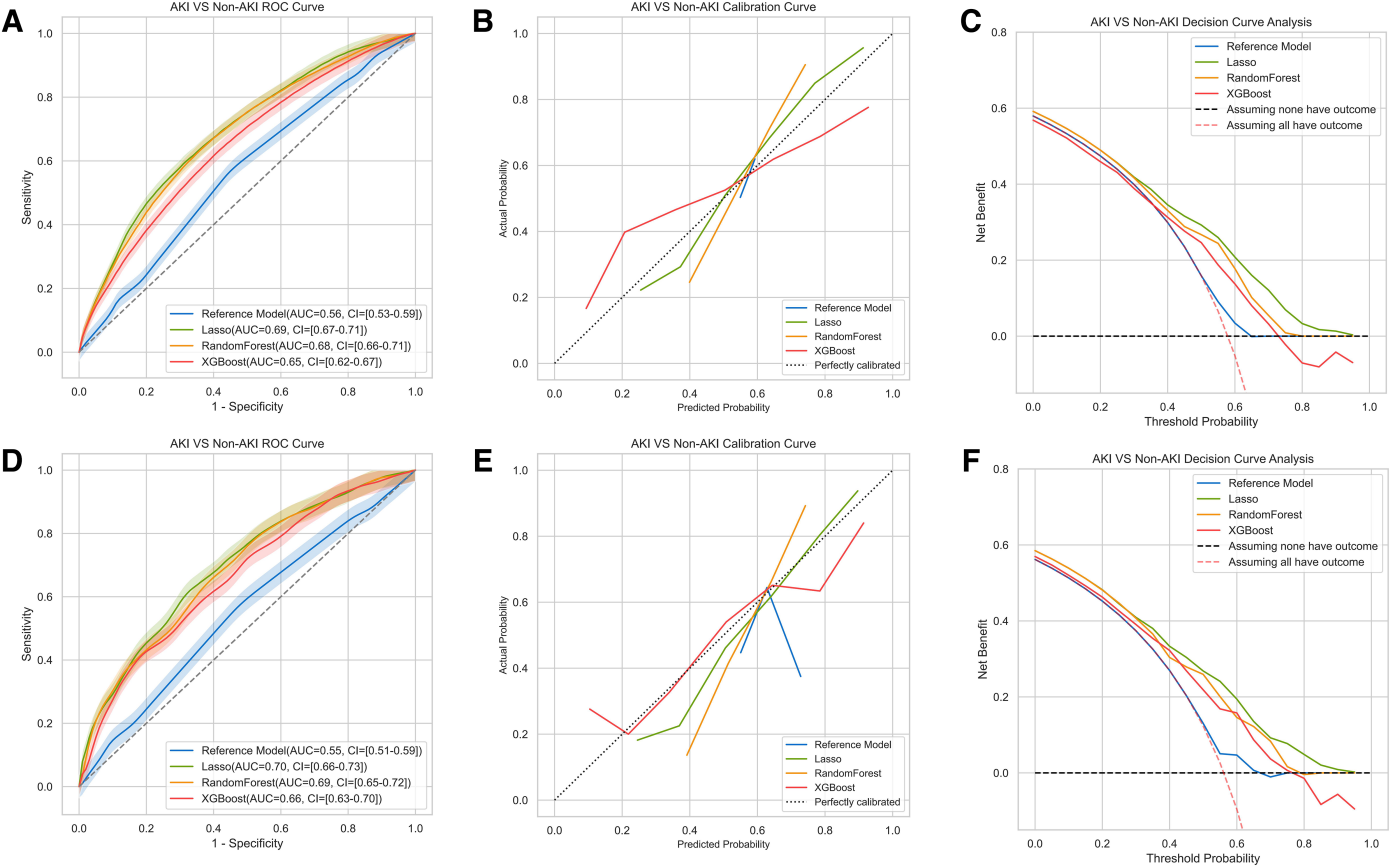
Comparison of AUCs, calibration curves and decision curve analysis among different machine learning models for AKI. The results of training set were shown in (**A–C**), testing set in (**D–F**). The model included the LLR model, the XGboost model and the Random Forest model. The reference model was AKICS score in (**A–C**).

In the machine learning algorithms of RF and XGboost, all the variables were used to predict postoperative all-stage AKI as input. In general, all three models including lasso logistic regression (LLR), RF and XGboost demonstrated varying but promising performance in predicting all stage AKI regarding the discrimination and calibration. Among the models, the lasso logistic regression model exhibited a slightly larger AUC of 0.69 (95% confidence interval [CI] 0.67–0.71) with an accuracy of 65% (95% CI, 63%–67%) in the training set (Figure 1A and Table 2). The AUCs of RF and XGboost were 0.68 (95% CI 0.66–0.71) and 0.65 (95%CI 0.62–67), respectively (Figure 1A). The AUCs of LLR, RF and XGboost in the testing set were 0.70 (0.66–0.73), 0.69 (0.65–0.72) and 0.66 (CI 0.63–0.70), respectively (Figure 1D). The discriminations of the three models were comparable, with the lasso logistic regression model marginally outperforming the other two. The three models accurately calibrated across the spectrum of all probabilities. The LLR model aligned with the 45-degree line the best (Figures 1B,E). Events tended to be overestimated at lower probabilities and underestimated at higher probabilities in LLR and RF models, while the trend was opposite in the XGboost model.

**Table 2:** Prediction performance of the reference, and machine learning models for all stages of postoperative CSA-AKI in patients underwent CPB assisted open heart valvular procedures.

	AUC	Accuracy	Sensitivity	Specify	F1	PPV	NPV	NRI	IDI
**Training set**									
Reference Model	0.56 (0.53–0.59)	0.60 (0.57–0.62)	0.95 (0.91–1.00)	0.08 (0.00–0.14)	0.74 (0.72–0.75)	0.60 (0.57–0.63)	0.34 (0.00–0.61)	0.00 (0.00–0.00)	0.00 (0.00–0.00)
Lasso	0.69 (0.67–0.71)	0.65 (0.63–0.67)	0.81 (0.76–0.87)	0.41 (0.35–0.48)	0.73 (0.71–0.75)	0.67 (0.63–0.70)	0.60 (0.55–0.67)	0.20 (0.14–0.25)	8.08 (6.79–9.25)
Random forest	0.68 (0.66–0.71)	0.64 (0.61–0.66)	0.88 (0.82–0.93)	0.29 (0.21–0.38)	0.74 (0.73–0.76)	0.64 (0.60–0.68)	0.63 (0.56–0.70)	0.14 (0.09–0.20)	4.14 (3.14–5.15)
XGBoost	0.65 (0.62–0.67)	0.63 (0.60–0.65)	0.75 (0.70–0.79)	0.45 (0.39–0.50)	0.70 (0.68–0.72)	0.66 (0.63–0.69)	0.55 (0.49–0.59)	0.17 (0.10–0.23)	11.39 (9.10–13.94)
**Testing set**									
Reference Model	0.55 (0.51–0.59)	0.56 (0.53–0.59)	0.93 (0.91–0.95)	0.08 (0.05–0.11)	0.70 (0.67–0.73)	0.57 (0.53–0.60)	0.46 (0.34–0.58)	0.00 (0.00–0.00)	0.00 (0.00–0.00)
Lasso	0.70 (0.66–0.73)	0.65 (0.62–0.68)	0.83 (0.80–0.86)	0.41 (0.36–0.46)	0.73 (0.70–0.76)	0.65 (0.61–0.68)	0.65 (0.60–0.71)	0.23 (0.17–0.30)	9.92 (7.81–12.03)
Random forest	0.69 (0.65–0.72)	0.63 (0.59–0.66)	0.90 (0.87–0.92)	0.28 (0.24–0.33)	0.73 (0.70–0.76)	0.62 (0.58–0.65)	0.68 (0.61–0.74)	0.17 (0.12–0.22)	5.45 (3.97–6.98)
XGBoost	0.66 (0.63–0.70)	0.62 (0.59–0.65)	0.77 (0.73–0.81)	0.42 (0.38–0.47)	0.69 (0.66–0.73)	0.63 (0.59–0.67)	0.59 (0.53–0.64)	0.18 (0.12–0.26)	12.70 (9.85–16.12)

The reference model was Cleveland Clinical score. AUC, area under the receiver operating characteristic curve; PPV, positive predictive value; NPV, negative predictive value; NRI, net reclassification improvement; IDI, integrated discrimination improvement.

In models for moderate to severe AKI, the AUC of the RF model (0.73, 95%CI 0.69–0.78) was numerically higher than that of the LLR model (0.69, 95%CI 0.64–0.75) and the XGboost (0.68, 95%CI 0.64–0.74) (Supplementary Table S2 and Appendix Figure S3A). However, all the models for stage 2 or stage 3 AKI were not calibrated well (Appendix Figure S3B). This may be due to the relatively low incidence of events.

### Model performance compared to previously published reference model

The previously reported AKICS was tested as the reference model for the prediction of all-stage AKI. It had an AUC of 0.56 (95%CI 0.53–0.59) and 0.55 (95%CI 0.51–0.59) in the training and testing sets, respectively (Figure 1A). Moreover, all three models demonstrated higher accuracy, negative predictive value, and positive predictive value in both the training set and testing set as compared to the reference AKICS score (Table 2). The net reclassification index (NRI) in the training set was 0.20 (95%CI 0.14–0.25), 0.17 (95%CI 0.10–0.23), and 0.14 (95%CI 0.09–0.20) in the LLR, XGboost and RF models, respectively. In the testing set, the LLR model had the largest NRI of 0.23 (95%CI 0.17–0.30) when compared with the reference AKICS score. XGboost had the best-integrated discrimination improvement (IDI) among all three models, with an IDI of 11.39 in the training set and 12.7 in the testing set compared to the AKICS. Both NRI and IDI were in favor of the newly constructed models (Table 2). Similarly, the newly developed models outperformed the reference Cleveland Clinical score in predicting moderate to severe AKI after cardiac surgery, all three models had higher IDI and NRI when compared with the reference Cleveland clinical score in predicting stage 2 or stage 3 AKI after cardiac surgery (Supplementary Table S2). Likewise, the DCA confirmed that the net benefit of newly developed models exceeded that of the reference model in both the training and testing sets throughout the threshold spectrum for all-stage AKI prediction (Figures 1C,F). For the prediction of moderate to severe AKI, the constructed models also out-performed the reference model across the full threshold range (Appendix Figure S3C).

### Interpretation and application of the predictive models

**Figure 2:**
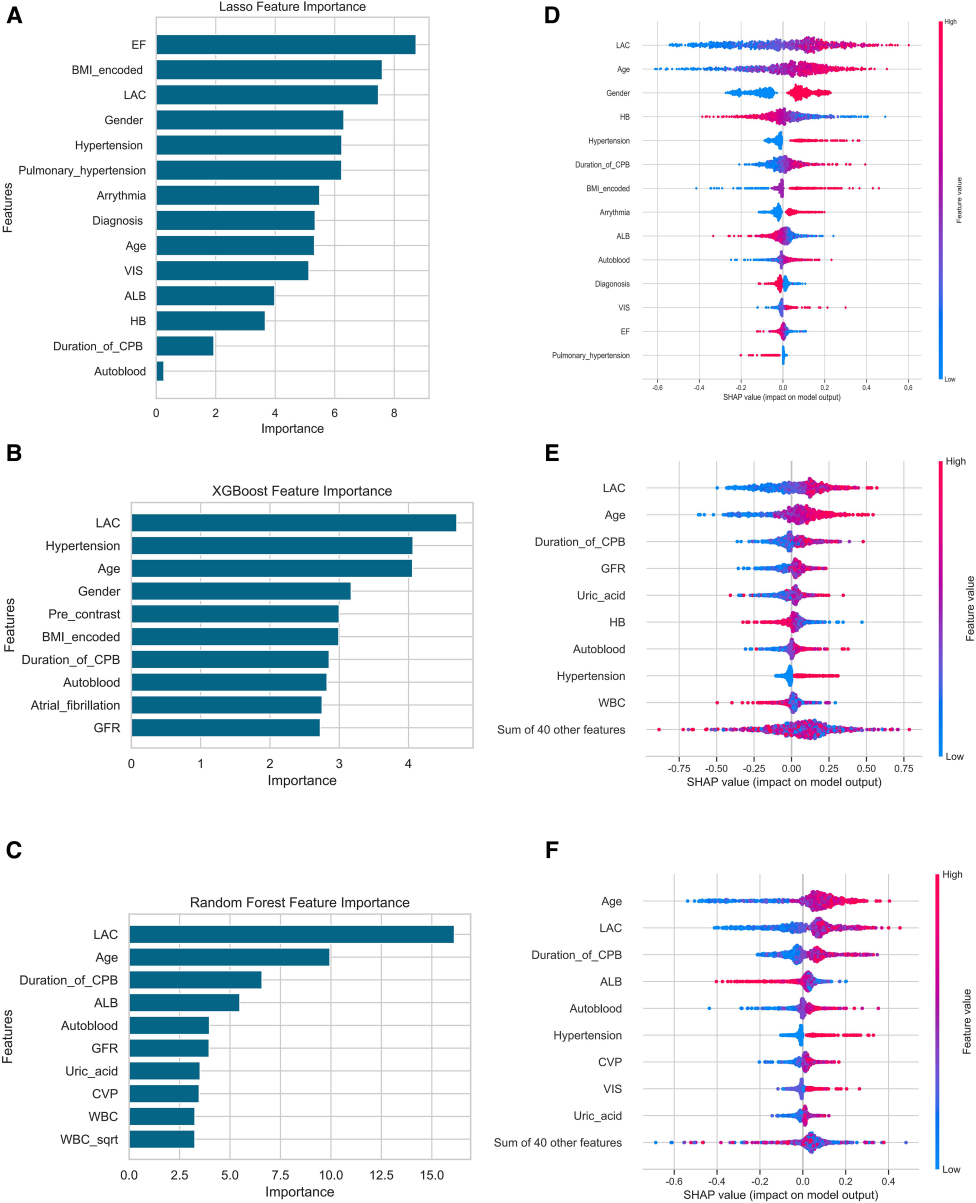
Importance matrix plot and SHAP summary plots of the LLR, the XGboost and the random forest model for AKI. (**A,D**) were the results of the LLR model, (**B,E**) were XGboost, (**C,F**) were the Random Forest model. Among the laboratory variables in the figure, only lactate was an intraoperative variable, others were baseline variables, and lactate was the highest level in the operation. Abbreviations: Lac, lactate; CPB, cardiopulmonary bypass; HB, hemoglobin, TBIL total bilirubin, bScr2 baseline serum creatinine, GFR glomerular filtration rate, ALB Albumin, CVP central venous pressure; WBC, white blood cell; VIS, vasoactive-inotropic score.

To quantify the importance of each input variable to the models, we performed an importance matrix plot and SHAP summary plot for all three models. In the importance matrix plot (Figures 2A–C), the highest level of lactate during the operation was the most important variable in all three models. Of the top 10 most important features, 4 were intraoperative variables in the LLR importance matrix plot, and 1 was postoperative variables in the RF importance matrix plot. The SHAP summary plots (beeswarm plots, Figures 2D–F) and the top 10 features were depicted to identify the features that influenced the prediction model most. The important features of LLR, RF, and XGboost were illustrated in order of importance from top to bottom. The plots demonstrated how high and low the values of the features were in association with the SHAP values. The higher the SHAP value was, the more likely to develop AKI. The plots also indicated if the features were risk or protective factors. If a greater feature value corresponded to a higher SHAP value, the feature should be considered a risk factor, and vice versa.

**Figure 3:**
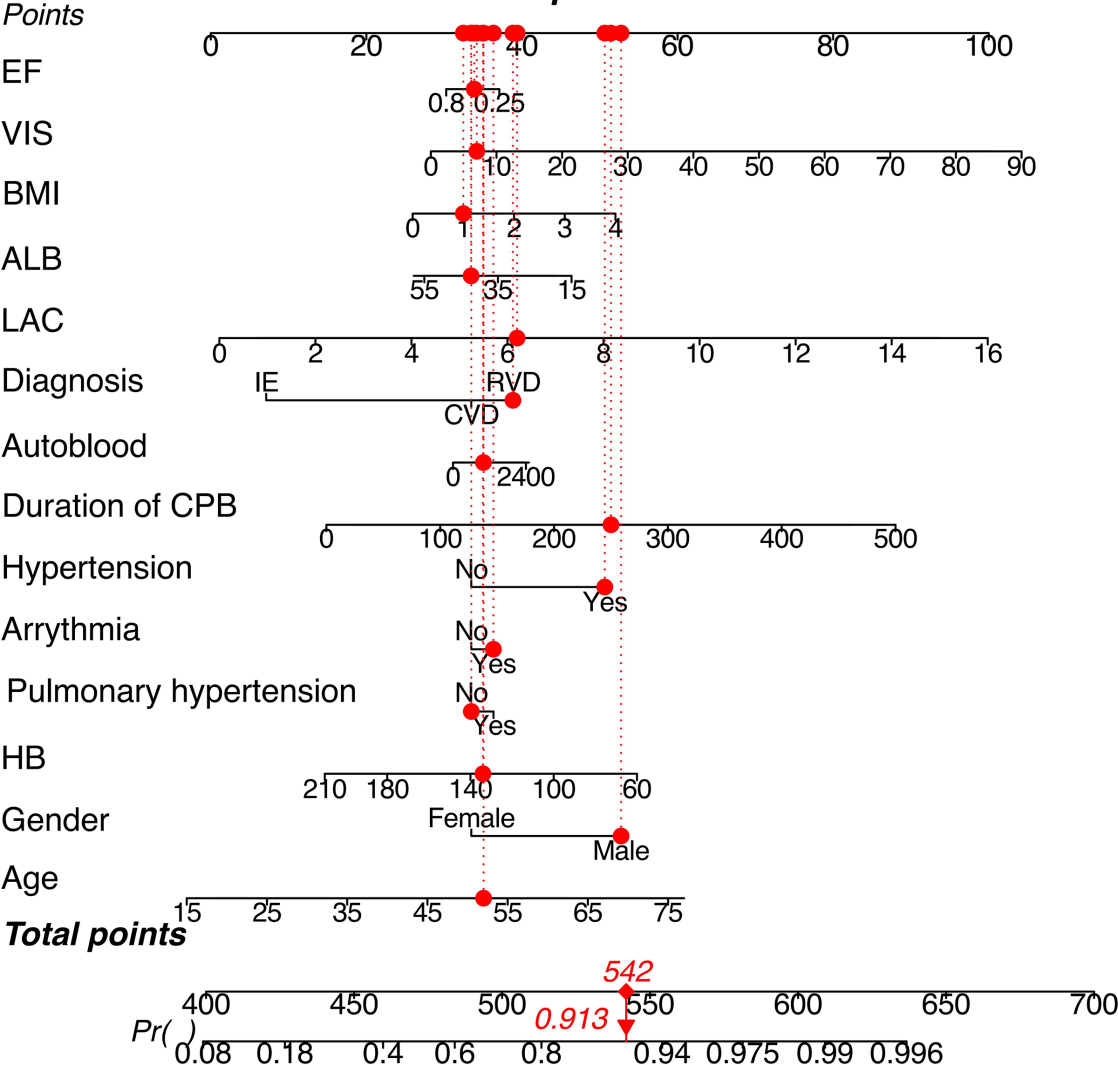
Risk-prediction nomogram in a patient with AKI after valvular cardiac surgery. Lactate was the highest level during CPB, autologous blood transfusion was the volume during CPB. HB was the latest value before operation. Abbreviations: LAC, lactate; HB, hemoglobin; ALB, albumin; VIS, vasoactive-inotropic score; EF, ejection fraction.

Finally, the independently associated risk factors from the best-performed lasso logistic regression model were used to develop an estimation nomogram to facilitate clinical use (Figure 3). The nomogram could assign the probability of AKI by summing the scores for each variable and positioning them on the total point scale. For instance, examining patient 230 in the database, the red points represent each variable's contribution to the score line. After adding the points to calculate the total score, and probability of AKI for this patient was determined to be 91.3%. In fact, this patient developed stage 2 AKI. Based on the nomogram, a dynamic web-based probability calculator was constructed to predict the incidence of AKI after cardiac surgery (https://anun.shinyapps.io/AKI-prediction/). The variance inflation factor of each variable from the best-performed lasso logistic regression model was less than 2, and the collinearity between variables was small.

## Discussion

In this retrospective cohort study, we developed and compared various prediction models using preoperative, intraoperative and early postoperative features to predict CSA-AKI following open heart valvular procedures. The lasso logistic regression model performed marginally better than the other two machine learning algorithms in predicting all-stage AKI and moderate to severe AKI. The newly developed models surpass the previously published reference models, i.e., the AKICS score and Cleveland Clinical score.

The prognosis of AKI varies based not just on etiology and clinical scenario but also on the degree of renal function impairment. Even stage I AKI was reported to be related to poor prognosis. In prior studies, even a minimal rise in serum creatinine was strongly related to long-term complications and increased mortality in cardiac surgery patients (24). As a transient disease state, stage I AKI can also be reversed by effective intervention, such as goal-directed fluid management. Thus, early recognition and initiation of effective AKI management are key to improving its prognosis (8). An accurate assessment of AKI risk can facilitate clinical decision-making and initiation of effective management, such as the KDIGO bundle (25, 26), to decrease the incidence and attenuate the severity of AKI after cardiac surgery.

Unfortunately, when recognition, diagnosis, and treatment of postoperative AKI are delayed or inadequate, it results in potentially avoidable increases in cost, other severe complications, and mortality (27). The models presently available to predict AKI have not effectively resulted in early diagnosis (8). The potential reason may be that the response of therapy varies in different risks of patients. For example, remote ischemic preconditioning did not improve clinical outcomes in patients undergoing elective on-pump CABG with or without valve surgery (28). However, among high-risk patients undergoing cardiac surgery, remote ischemic preconditioning significantly reduced the rate of acute kidney injury and the use of renal replacement therapy (29, 30). So, it is paramount to screen high-risk patients for specific treatment. Additionally, there is heterogeneity among different populations and different cardiac surgery types.

Existing predictive models of AKI mainly focus on all types of cardiac surgery. The risk of incident AKI varies in different cardiac surgical procedures. The risk among patients undergoing combined coronary artery bypass grafting (CABG)-valve procedures is the greatest, followed by valve replacement surgery and lowest with CABG alone (31). Therefore, many factors should be considered in the development of an AKI model.

Established risk scores for AKI prediction following cardiac surgery mainly focused on AKI requiring dialysis and severe AKI, and there were some widely accepted clinical risk scores developed to predict RRT-AKI, including the Cleveland Clinical score, SRI score, Mehta score and a score developed by Pannu et al. (10, 11, 13, 32). Some clinical scores aimed to predict severe AKI, including the Multicenter Study of Perioperative Ischemia Score, the Acute Kidney Injury After Cardiac Surgery Score, the AKICS score and the cardiac surgery-associated-severe acute kidney injury score (12, 23, 33, 34). These risk scores varied with different definitions of AKI, and only two risk scores incorporated post-operation variables, i.e., the AKICS score and the cardiac surgery-associated severe acute kidney injury score (35). Besides, these models had substantial flaws, such as similarity among models, rarely being externally validated, being heterogenous in AKI definitions, having a high risk of bias, being based on small cohorts, and ignoring intra- and postoperative variables (35). No impact analysis studies in which the clinical application of a model was utilized to underpin or influence interventional strategies for prevention or treatment had been documented.

Increasingly, machine learning and other potentially potent techniques are being reported to identify AKI predictors. In terms of predicting AKI following cardiac surgery, the models developed by machine learning performed better than traditional risk scores. Lee and colleagues initially adopted a machine learning approach to predict AKI following cardiac surgery. Among the developed models, the XGboost model showed the best performance (36). However, in the current study, the traditional logistic regression model performed marginally better at predicting AKI than machine learning models. The sample size in our study was roughly five times than that of Lee and colleagues, which could partially explain the discordance in results. Demirjian et al. have recently reported a new prediction model for adult patients undergoing coronary artery bypass graft, valve, or aorta surgery. This study had a substantial sample size, rigorous statistical analyses, and conducted external validation. However, important features believed to be closely associated with CSA-AKI, including the use of cardiopulmonary bypass, intra-aortic balloon pump, and the length of the aortic cross-clamp, were omitted from the model (37). As indicated in the importance matrix plots and the SHAP summary plots, half of the top 10 features in our final model were intraoperative variables. Focusing solely on the preoperative variables while neglecting intra- and post-operative features is likely to dramatically compromise the accuracy and application of AKI prediction models.

The previously published risk scoring models, AKICS and Cleveland Clinical score performed poorly in our cohort. This could be largely attributable to the disparity in the models' derivation population. The widely used reference models were developed from Europe and North American cardiac surgery patients. They differed in race and ethnicity from the cohorts in our study. Our cohort consisted of Chinese valvular procedure patients who predominantly suffered from rheumatic heart disease. This patient population is younger, healthier, and has fewer comorbidities than the population undergoing valve surgery in Europe and North America who are mainly affected by the degenerative valvular disease (38). When generalizing a prediction model, heterogeneity in model performance across populations, settings, and periods should be considered (39).

A recently developed model was found to be useful in predicting the severity of AKI in Chinese patients undergoing on-pump cardiac surgery (40). This study featured a large sample size and external validation. However, AKI was diagnosed with the AKIN classification, not the KDIGO criteria. Furthermore, the incidence of AKI was drastically different in derivation (14.7%) and validation (42.3%) cohorts. This huge discordance in event rates may result in differing predictor value distributions and heterogeneity in the patient population. Therefore, it was no surprise that the baseline characteristics of patients in the derivation and external validation cohorts were highly disparate. Moreover, postoperative variables were not included in the final model. Therefore, the model's generalizability was limited.

Our study has several limitations. First, our analysis was based on data from a single center, and the external validity needs to be confirmed. To evaluate the generalizability of our risk scoring system, prospective multicenter validation studies in different settings are warranted. In fact, we are planning a multicenter external validation of our model in the Chinese population. Second, we intend to include early postoperative factors into the model to improve its predictive accuracy. In our current study, only CVP at ICU admission data was available, and Lasso logistic regression did not ultimately pick this variable. Third, it is uncertain whether incorporating the developed prediction model into clinical practice to facilitate decision-making and guide perioperative management will improve patient prognosis. This needs to be verified in future randomized clinical trials.

## Conclusion

Among the Chinese population undergoing CPB-assisted valvular cardiac surgery, a prediction model based on perioperative variables demonstrated good predictive performance for all stage AKI after surgery.

## Data Availability

The raw data supporting the conclusions of this article will be made available by the authors, without undue reservation.
