# Supplementary material for: Perioperative parameters-based prediction model for acute kidney injury in Chinese population following valvular surgery
Source: Front Cardiovasc Med. 2023 Mar 7;10:1094997. doi: 10.3389/fcvm.2023.1094997 (PMC10028074; doi:10.3389/fcvm.2023.1094997)

Supplementary material

[Figure S1: Feature selection by LASSO. 2](#_Toc127645325)

[Figure S2: Flowchart of patients. 2](#_Toc127645326)

[Figure S3: Comparison of AUCs, Calibration curves and Decision curve analysis among different machine learning models for stage 2 and stage 3 AKI. The reference model was Cleveland Clinical score. 3](#_Toc127645327)

[eTable1: Candidate variables 4](#_Toc127645328)

[eTable 2: Prediction performance of the reference, and machine learning models in postoperative CSA-AKI in patients underwent CPB assisted open heart valvular procedures. The reference model was Cleveland Clinical score. 5](#_Toc127645329)

# Figure S1: Feature selection by LASSO.

# Figure S2: Flowchart of patients.

# Figure S3: Comparison of AUCs, Calibration curves and Decision curve analysis among different machine learning models for stage 2 and stage 3 AKI. The reference model was Cleveland Clinical score.

# eTable1: Candidate variables

| (1) Demographics, including age, gender, and body mass index (BMI); |
| --- |
| (2) Comorbidities and medical history, including history of cardiac surgery (either open-heart or percutaneous mini-invasive procedure), angina, stroke, myocardial infarction (MI), chronic obstructive pulmonary disease (COPD), pulmonary hypertension (PAH), arrhythmia, atrial fibrillation (AF), diabetes with insulin, hypertension, liver disease, smoking status, drinking status, and baseline renal (eGFR, i.e., estimated glomerular filtration rate [eGFR] calculated by CKD-EPI formula) and heart function (left ventricular ejection fraction, and classification of New York Heart Association [NYHA]); |
| (3) Diagnosis, and surgery procedure; |
| (4) Baseline laboratory tests, including hemoglobin, white blood cell counts, albumin, total bilirubin, uric acid, serum creatinine and prothrombin time; these variables were the latest value before operation; |
| (5) Preoperative medication, including contrast agent, nephrotoxic antibiotics (i.e., vancomycin), angiotensin-converting enzyme inhibitors (ACEI)/angiotensin receptor antagonist (ARB), levosimendan, and non-steroidal anti-inflammatory drugs (NSAID); |
| (6) Preoperative use of intra-aortic balloon pump; |
| (7) Operation related predictors, including duration of cardiopulmonary bypass (CPB) and aortic-clamping time, serum glucose, lowest temperature, minimum hematocrit, volume of hydroxyethyl starch volume of autologous blood transfusion, fluid balance per kilogram of body weight, maximum vasoactive/inotropic score, highest level of lactic acid; |
| (8) Measurements of central venous pressure (CVP) at admission to the ICU. |

# eTable 2: Prediction performance of the reference, and machine learning models in postoperative CSA-AKI in patients underwent CPB assisted open heart valvular procedures. The reference model was Cleveland Clinical score.

| **Outcome and Model** | **AUC** | **Accuracy** | **Sensitivity** | **Specify** | **F1** | **NPV** | **PPV** | **NRI** | **IDI** |
| --- | --- | --- | --- | --- | --- | --- | --- | --- | --- |
| Reference Model | 0.48(0.43-0.50) | 0.93(0.91-0.94) | 0.00(0.00-0.00) | 1.00(1.00-1.00) | 0.00(0.00-0.00) | 0.93(0.91-0.94) | 0.00(0.00-0.00) | 0.00(0.00-0.00) | 0.00(0.00-0.00) |
| Lasso | 0.69(0.64-0.75) | 0.93(0.91-0.94) | 0.04(0.00-0.08) | 1.00(1.00-1.00) | 0.07(0.00-0.15) | 0.93(0.92-0.94) | 0.75(0.00-1.00) | 0.04(0.00-0.08) | 5.99(3.53-8.47) |
| Random forest | 0.73(0.69-0.78) | 0.93(0.91-0.94) | 0.01(0.00-0.03) | 1.00(1.00-1.00) | 0.02(0.00-0.05) | 0.93(0.91-0.94) | 0.48(0.00-1.00) | 0.01(0.00-0.03) | 4.76(3.18-6.48) |
| XGBoost | 0.68(0.64-0.74) | 0.92(0.91-0.94) | 0.05(0.01-0.08) | 0.99(0.99-1.00) | 0.08(0.03-0.14) | 0.93(0.92-0.94) | 0.40(0.14-0.79) | 0.04(0.01-0.08) | 5.80(2.86-8.73) |

Abbreviations: AUC Area under the receiver operating characteristic curve, PPV positive predictive value, NPV negative predictive value, NRI net reclassification improvement, IDI integrated discrimination improvement


Supplement: Supplementary file 1 [file Datasheet1.docx]
